# Selective Targeting of Tumorigenic Cancer Cell Lines by Microtubule Inhibitors

**DOI:** 10.1371/journal.pone.0004470

**Published:** 2009-02-13

**Authors:** Newaj M. Abdullah, Gus R. Rosania, Kerby Shedden

**Affiliations:** 1 Department of Pharmaceutical Sciences, University of Michigan College of Pharmacy, Ann Arbor, Michigan, United States of America; 2 Department of Statistics, University of Michigan, Ann Arbor, Michigan, United States of America; 3 Michigan Alliance for Cheminformatics Exploration (MACE), Ann Arbor, Michigan, United States of America; University of the Western Cape, South Africa

## Abstract

For anticancer drug therapy, it is critical to kill those cells with highest tumorigenic potential, even when they comprise a relatively small fraction of the overall tumor cell population. We have used the established NCI/DTP 60 cell line growth inhibition assay as a platform for exploring the relationship between chemical structure and growth inhibition in both tumorigenic and non-tumorigenic cancer cell lines. Using experimental measurements of “take rate” in ectopic implants as a proxy for tumorigenic potential, we identified eight chemical agents that appear to strongly and selectively inhibit the growth of the most tumorigenic cell lines. Biochemical assay data and structure-activity relationships indicate that these compounds act by inhibiting tubulin polymerization. Yet, their activity against tumorigenic cell lines is more selective than that of the other microtubule inhibitors in clinical use. Biochemical differences in the tubulin subunits that make up microtubules, or differences in the function of microtubules in mitotic spindle assembly or cell division may be associated with the selectivity of these compounds.

## Introduction

The aggressiveness of different kinds of tumor cells derived from human patients can be assessed in terms of their tumorigenic potential in mouse xenograft models. For example, tumorigenic potential in mouse xenografts has recently been used to define the cancer “stem cells”, which presumably correspond to the subpopulation of malignant cells that drive the formation and growth of the tumor [1]. Accordingly, it has been postulated that some cancers are composed of a heterogeneous collection of cells, only a minority of which are capable of forming new tumors [2]. These cells can be enriched from heterogeneous tumor cell populations on the basis of their expression of cell-surface markers. In breast tumors, for example, cells co-expressing high levels of CD44 and epithelial specific antigen (ESA) and low levels of CD24 are the tumor initiating cells [2]. Likewise, in colon and brain cancer, subpopulations of cells expressing high levels of CD133 (PROML1) initiate the tumors [3], [4]. Most importantly, upon transplantation into immunocompromised mice, tumor-initiating cells can fully reconstitute a tumor with heterogeneity reminiscent of the original tumor [2]–[4]. Although the concept of a cancer “stem cell” is still controversial, from a therapeutic standpoint, anticancer agents directed against tumorigenic cancer cells may be the most effective at eradicating tumors.

The drug discovery and development sector of National Cancer Institute (NCI), the Developmental Therapeutics Program (DTP), has utilized a panel of 60 human tumor-derived cell lines to screen the chemotherapeutic potential of more than 75000 compounds [5], [6]. This panel of 60 cell lines is commonly known as “NCI60 cell lines.” The cell lines represent various leukemias, melanomas and cancers of the lung, colon, brain, ovary, breast, prostate and kidney [5]. Apart from their use in drug screening, the tumorigenic potential of these cell lines has been measured by xenotransplanting these cells into immunocompromised mice and assessing their ability to form new tumors [6]. Different cell lines in the NCI60 panel display a range of tumorigenic potentials upon transplantation into immunocompromised mice. The tumorigenic potential has been recorded as each cell line's “take-rate.”

As a hypothesis, differences in tumorigenic potential among the NCI cancer cell lines may reflect variations in proliferative activity and tumor-initiating characteristics of the actual cancer cells as they exist in the tumors of cancer patients. Thus, NCI60 cell lines demonstrating high take rate may be more representative of tumor-initiating cancer cells found *in situ*. Here, we identify compounds from the DTP database that are most active against cell lines with the highest take rate, and proceed to establish a putative mechanism of action for these compounds by performing structure-activity relationship studies, and comparing them to standard anticancer agents whose mechanism of action is known. In addition, differences in tumorigenic potential and responsiveness to these agents are shown to be related to differences in gene expression between NCI60 cell lines with high and low tumorigenic potentials, as well as to gene expression markers of tumorigenic cancer cells.

## Results

### Identification of selectively cytotoxic compounds

**Figure 1:**
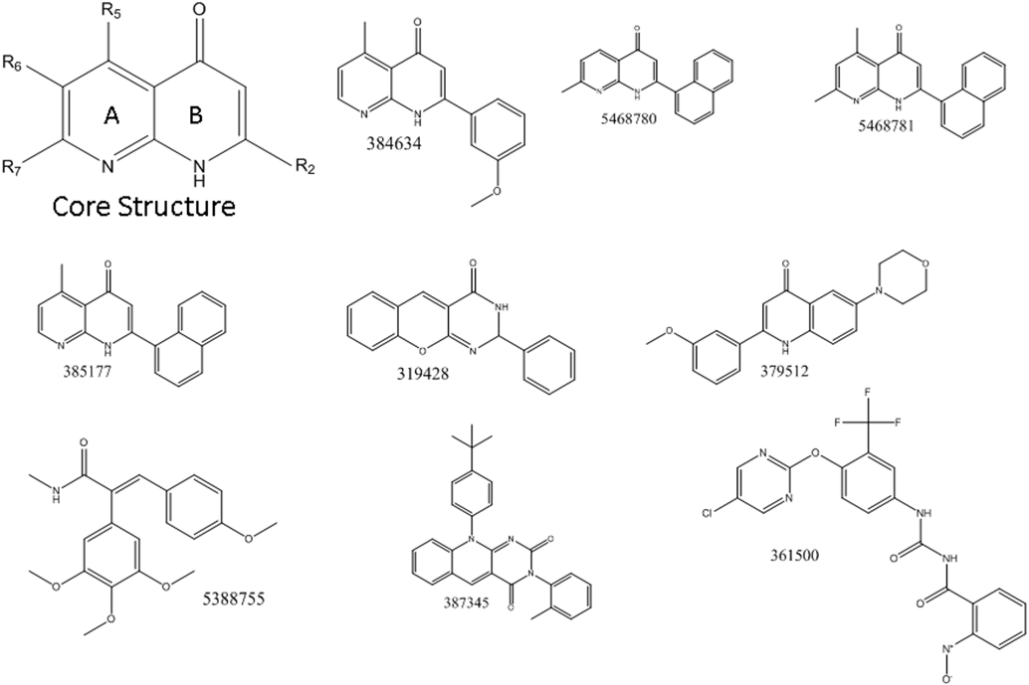
Structures of nine selectively cytotoxic compounds and the core structure shared by four of these compounds.

**Figure 2:**
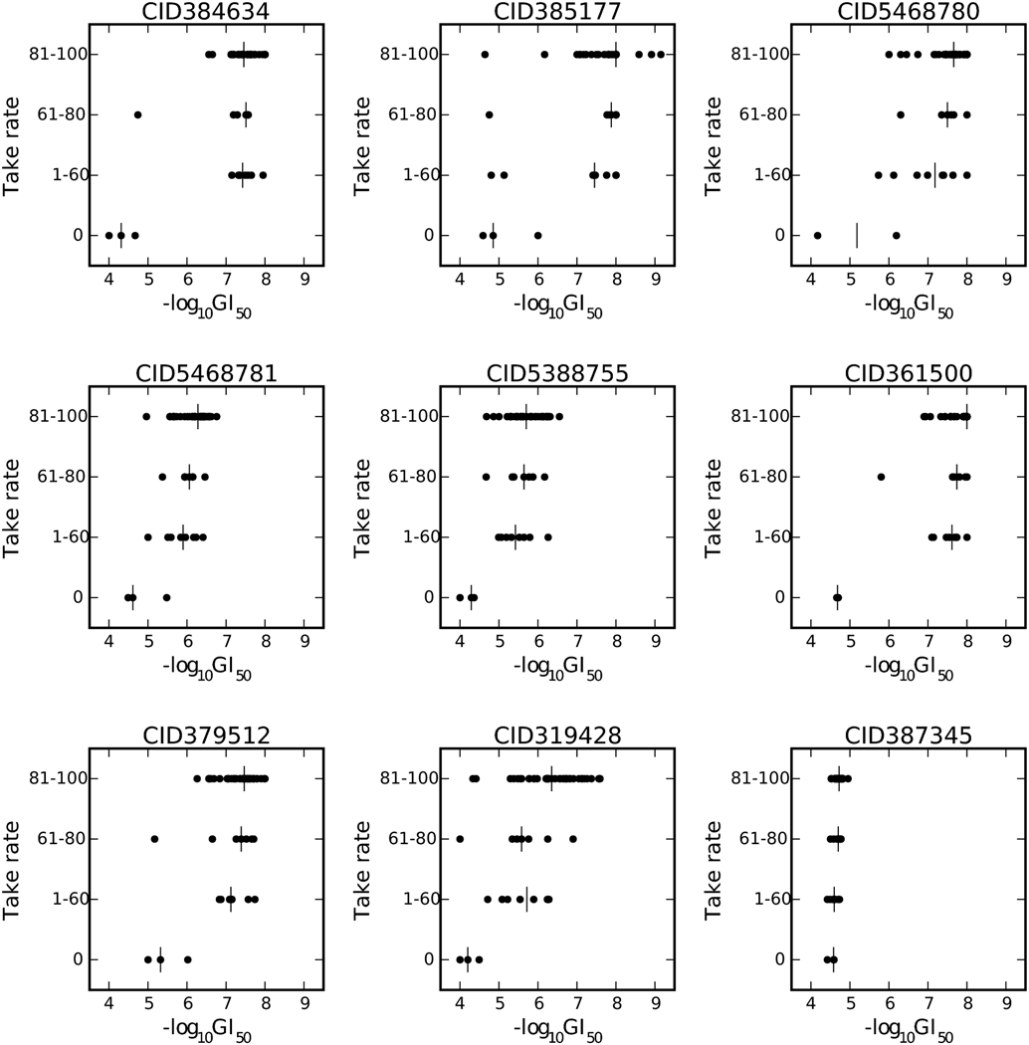
Scatterplot of cytotoxic activity (−logGI50) for the nine compounds identified in our virtual screen as showing cytotoxic activity, in relation to the four categories of tumorigenic potential.

Growth inhibitory activity in the DTP collection of chemical agents as represented by −logGI50 can be compared to the four categories of take-rate using Pearson correlation coefficients. Using this approach, nine compounds having correlation coefficient greater than 0.5 in magnitude were identified out of 34,909 compounds tested (Figure 1). All nine correlation coefficients were positive, indicating that these agents were more active at inhibiting cell growth in the most tumorigenic cell lines (Figure 2). Because the expected number of compounds out of 34,909 having a correlation coefficient exceeding 0.5 in magnitude by chance is 0.7 with a 95^th^ percentile of two compounds, it is very unlikely that two or more of these nine compounds are false positives.

**Figure 3:**
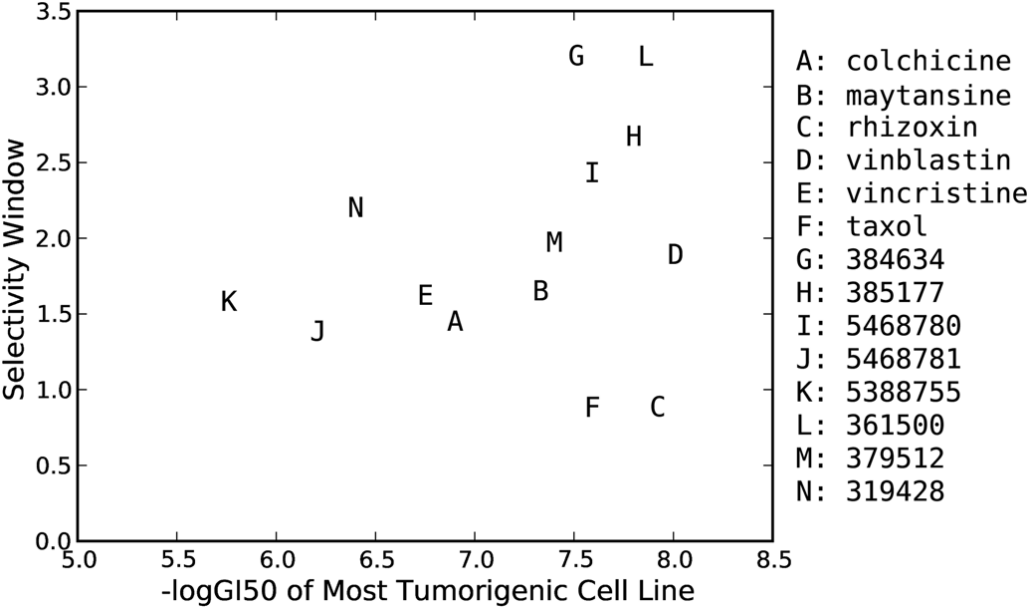
Selectivity windows for eight compounds identified in our virtual screen (G–N) and several standard anticancer agents having antimicrotubule activity (A–F).

None of the standard anticancer agents in the DTP database surpass these nine compounds in terms of selective cytotoxic activity against the most tumorigenic cell lines. The greatest correlation coefficient observed among the standard anticancer agents is 0.47 for vinblastine, which is an antimitotic agent. In fact, antimitotic agents are the only mechanistic class showing consistent non-negligible positive correlation with take-rate. Despite their positive correlation coefficients, none of the antimitotic standard anticancer agents show correlation coefficient greater than 0.5, suggesting that the nine compounds identified in our correlation analysis may be uniquely selective against the most tumorigenic cell lines. Several of these nine compounds exhibit a wide selectivity window with difference in −logGI50 between tumorigenic and non-tumorigenic cell lines of two or more. Compounds 384634, 385177, 5468780, 361500 and 379512 are comparable to all of the standard antimitotic agents in regards to their cytotoxicity; however, their selectivity window is much wider (Figure 3).

### Inhibition of tubulin polymerization as possible mechanism of action

The compounds identified point to a major structure-activity relationship class: four of the compounds identified share a core naphthyridin structure (see Figure 1). Three of these compounds (385177, 5468780, 5468781) are structurally related, through the presence of a naphthelene group at position R_2_. These structures differ from each other based only on the positioning of one or two methyl group on the A ring: compounds 385177 and 5468780 contain a methyl group at positions R_5_ and R_2_, respectively, while compound 5468781 contains two methyl groups at positions R_5_ and R_2_. The other compound (384634) differs from the three previously mentioned compounds because the group 3′-methoxy substituted benzene ring substitutes the naphthalene group at position R_2_. This compound also contains a methyl group at position R_5_ on ring A. The presence of the core structure common to all the compounds in this group suggests that it may play a cornerstone role in the mechanism of action for this cohort of compounds.

In order to identify a possible mechanism of action, the nine compounds were clustered together with the 168 standard anticancer agents using the 881 key CACTVS fingerprints. Cutting the dendrogram at a Tanimoto coefficient of 0.7, five of the nine compounds are clustered with nine standard anticancer agents including various antitubulin agents such as vinblastine and vincristine. Subsequent analysis of the scientific literature revealed that many of our compounds do indeed inhibit polymerization of tubulin *in vitro*. Compound 384634 has been synthesized and has shown to demonstrate antitublin activity in a tubulin polymerization assay [11]. Likewise, isosteres of compound 385177, 5468780 and 5468781 potently inhibit tubulin polymerization [12]. It is highly plausible that compound 379512 is an antitubulin agent as well, because a number of compounds containing the 2-phenyl-quinolone ring structure have been synthesized and exhibit tubulin polymerization [13]–[17]. Compound 5388755 is almost structurally identical to Combretastatin A-4, which is a very potent antitubulin agent [18].

COMPARE analysis [19] was performed to further characterize the mechanism of action of the compounds. In COMPARE, a correlation coefficient of 0.6 is generally taken to indicate evidence for similar mechanisms of action between the tested and reference compounds. The higher the correlation coefficient, the more likely it is that the compounds share the same intracellular target [9]. The correlation coefficient of the COMPARE computations for the eight most potent compounds and the antimitotic standard anticancer agents reveals several compounds showing high correlations with microtubule inhibitors colchicine, maytansine, vinblastine and vincristine (Table 1). None of these compounds show similarity to any of the agents from other mechanistic classes such as topoisomerase inhibitors, alkylating agents and DNA/RNA antimetabolites (data not shown). None of the compounds exhibit strong correlation with taxol, which is an antimitotic agent that acts by stabilizing microtubules.

**Table 1:** COMPARE analysis of eight compounds to various agents from the antimitotic activity class.

Seed	Colchicine	Maytansine	Rhizoxin	Taxol	Vinblastine	Vincristine
384634	0.69	0.73	0.51	0.36	0.85	0.70
385177	0.50	0.42	0.41	0.32	0.51	0.36
5468780	0.60	0.62	0.22	0.32	0.63	0.58
5468781	0.55	0.59	0.31	0.38	0.59	0.46
319428	0.27	0.37	0.24	0.27	0.53	0.35
361500	0.75	0.72	0.58	0.34	0.77	0.56
5388755	0.67	0.70	0.60	0.44	0.63	0.60
379512	0.41	0.43	0.32	0.28	0.29	0.65

### Antitubulin activity parallels selective cytotoxicity

**Figure 4:**
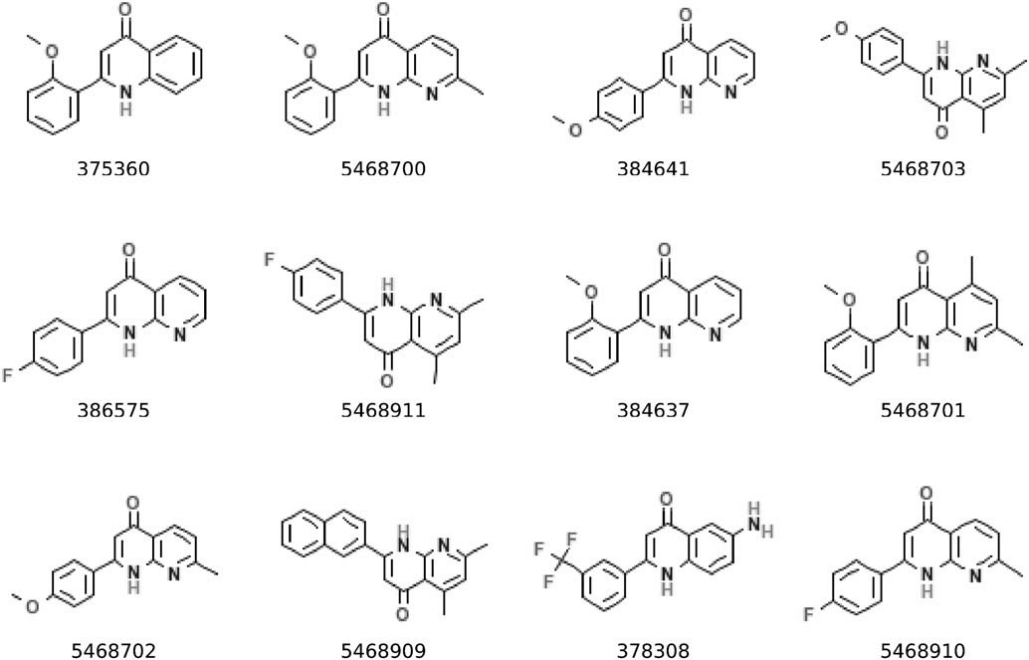
Structures of compounds that are structurally related to the nine compounds identified in our virtual screen, but that do not inhibit microtubules or cell growth.

**Figure 5:**
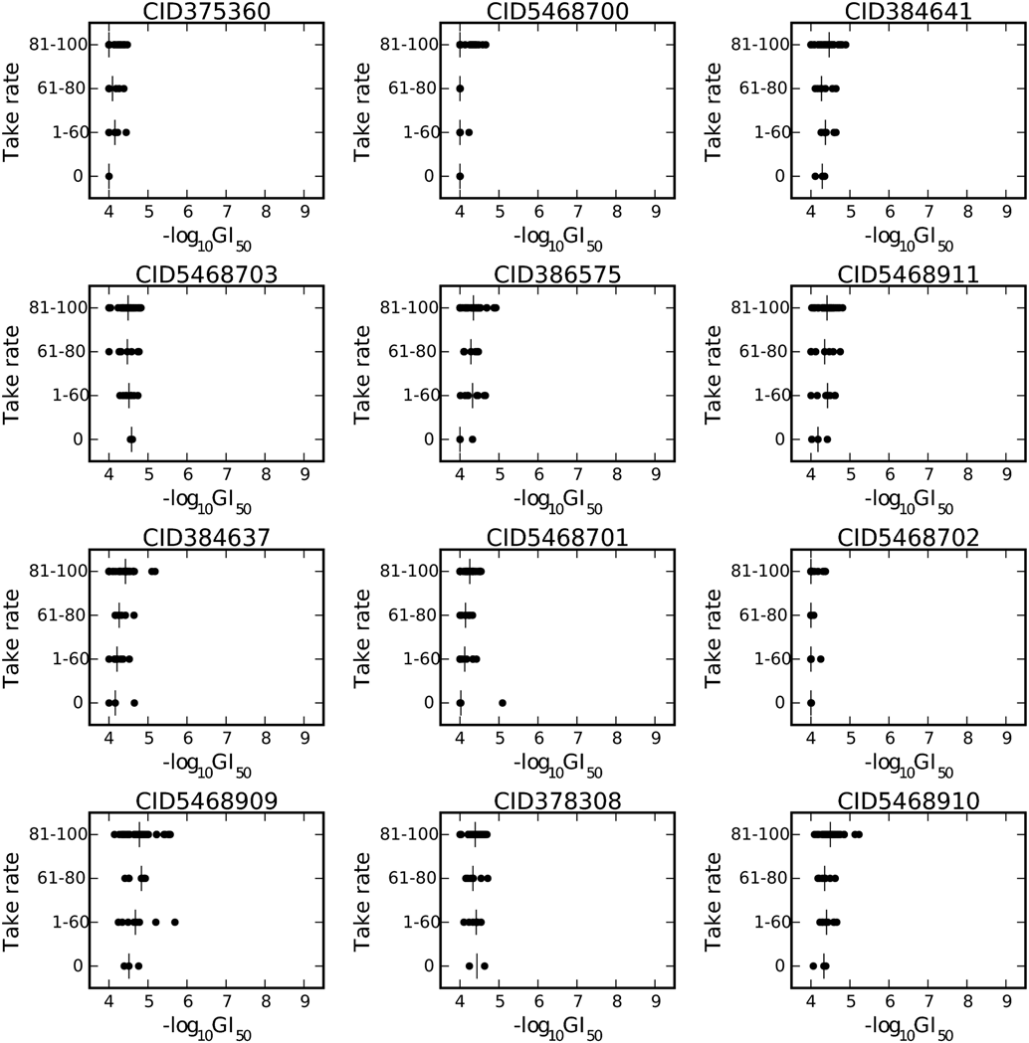
Scatterplots of the cytotoxicity of several compounds lacking microtubule inhibitory activity, in relation to the four categories of tumorigenic potential.

In order to identify the role of antitubulin activity in generating selective cytotoxicity, we identified twelve additional DTP compounds (Figure 4) that are structurally related to some of the nine compounds we identified in our correlation analysis but that lack antitubulin activity [11]–[15]. If antitubulin activity confers selective cytotoxicity, these compounds with no antitubulin activity should demonstrate no selective cytotoxicity. The scatterplot comparing the association between cytotoxicity and take-rate for these twelve compounds indicates that none of these compounds show selective cytotoxicity (Figure 5), and they are largely inactive in the cell growth inhibition assay.

### Gene expression analysis

A number of previous research studies have identified CD44, CD24, and CD133 (PROML1) as being markers for tumorigenic potential or stem-cell-like characteristics, with CD44 and CD133 being relatively highly expressed in tumorigenic lines, and CD24 being expressed at low levels. Thus, we searched for specific genes whose expression may be related to the selective cytotoxic activity of the compounds identified. For this purpose, transcriptional profiling data was mined for genes whose expression across the cell lines correlates with tumorigenic potential. In this data set, we found that take rate is independent of PROML1, CD44, and the log ratio CD44-CD24 in the NCI60 cell lines. We also analyzed the expression of twenty or so different tubulin isotypes (alpha, beta, and gamma) and found no correlation with take rate.

Although candidate tumorigenicity marker genes PROML1, CD44, and CD44-CD24 were not associated with take-rate, we did identify genes expressed at substantially higher levels in the more tumorigenic cell lines. On the U95A array platform, a transcription factor (DBP), an integrin (ITGA6, two probe sets), and a membrane skeletal protein (ADD3, two probe sets) followed this pattern of expression. Six named genes and two unnamed genes are expressed at substantially higher levels in the less tumorigenic as compared to the more tumorigenic cell lines on the U95A array platform: PTGIS, JAK1, MGC5560, XPC, NRG1, and SULF1. On the U133A/B platform TMEM18, ACACB, and GMCL1 were positively associated with tumorigenic potential, along with two unnanotated probesets (229930_at and 230312_at). No negative associations meeting our selection criteria were identified on the U133A/B array platform. The functional significance of putative stem cell marker genes in relation to tumorigenicity or increased sensitivity to microtubule inhibitors is not clear.

## Discussion

By data mining the DTP archive, we are able to identify compounds that are preferentially toxic against the most tumorigenic of the NCI60 cell lines, based on the take rate of the cell lines in a mouse xenograft model. We also established that the activity of these compounds was not correlated to the expression of cell surface stem cell markers reported in the literature. Nevertheless, tumorigenic potential is the most important functional relationship between the most aggressive tumor cells and *in vitro* model for drug screening. Therefore, the anticancer agents identified based on their activity against the most tumorigenic cell lines may be considered as candidate anticancer agents that are specifically directed against subpopulations of cancer cells that drive the growth of tumors.

One of these agents (384634) has been found to inhibit microtubule polymerization. Likewise, isosteres of three of our agents (385177, 5468780, 5468781) have also been shown to inhibit microtubule polymerization, suggesting a single mechanism of action. Interestingly, Compound 5388755 is structurally related to the potent antitubulin agent Combretastatin A-4. It is also possible that compound 379512 acts by inhibiting tubulin polymerization because several different agents containing the quinolone ring structure have demonstrated antitubulin activity. COMPARE analysis corroborates the similarities between the anticancer agents identified here and various different microtubule inhibitors. With the exception of compound 319428, all of our compounds show strong similarity with colchicine, maytansine, vinblastine and vincristine. None of our compounds show significant relationship to taxol, which acts by stabilizing microtubules.

From our analysis, antitubulin activity is likely to be responsible for selective cytotoxicity against tumorigenic cell lines. A select number of structurally related compounds with no antitubulin activity were analyzed for their pattern of cytotoxicity toward NCI60 cell lines. None of these compounds demonstrated selective cytotoxicity. In fact, most of these compounds were inactive. Together with their antitubulin activity, the selectivity of our compounds toward highly tumorigenic cell lines suggests that microtubules of tumorigenic and non-tumorigenic cell lines may differ. Interestingly, no difference in tubulin gene expression level was observed between highly tumorigenic and non-tumorigenic cell lines. It is plausible that observed selective cytotoxicity is not due to difference in tubulin gene expression but rather a result of differences in post-translational modifications (PTMs) [20]. Recently, various experimental results have supported the notion that tubulin PTMs lead to the functional diversity of microtubules. Many tubulin PTMs have been identified including detrysosination, glutamylation, glycylation, acetylation phosphorylation and palmitoylation [20]–[22]. Differences in tubulin isotype expression and PTMs have been associated with cell differentiation and developmental transitions [23]–[25]. Because microtubules are key to mitotic spindle assembly and cell division, differences in mitotic spindle structure and function between tumorigenic and nontumorigenic cell lines may be associated with the selectivity of these compounds.

In conclusion, we have identified a family of microtubule inhibitors that are mostly toxic against tumorigenic cell lines. Established cancer cell lines demonstrating high tumorigenicity in xenograft models may capture some properties of cancer cell subpopulations that are responsible for initiating and spreading the tumors. Therefore, we propose that this family of microtubule inhibitors, or related compounds with similar selectivity characteristics, should be considered as prime candidates for further evaluation as anticancer agents.

## Materials and Methods

### Primary data

The compound growth inhibition data was obtained from the NCI 60 cell line antitumor screen. The growth inhibitory activity of each compound corresponds to the molar drug concentration required to cause 50% growth inhibition (GI50). Most assays use a maximum concentration of 0.0001 M (the cell line screen and GI50 parameter are described in [7]). For microarray gene expression analysis, we used five publicly-available data sets for the NCI60 cell lines: triplicate experiments using the Affymetrix U95A platform provided by Novartis, a single U95A data set provided by GeneLogic, and a single Affymetrix U133A/B data set provided by GeneLogic. The GI50 data were obtained from http://dtp.nci.nih.gov/docs/cancer/cancer_data.html and all gene expression data were obtained from http://dtp.nci.nih.gov/mtargets/download.html. All gene expression and GI50 assay data are analyzed on the log scale.

### Rating of tumorigenic potential

The NCI60 cell lines have been experimentally evaluated for tumorigenic potential by transplantation of the cell lines into immuno-compromised mice. The experiments and results are provided in the Anticancer Drug Development Guide [6]. For different cell lines, these data are given either quantitatively or qualitatively, or sometimes as ranges, as the “take rate,” or proportion of attempted implants that yielded a tumor. We converted the take rate data into four ordered categories for analysis: 0 for no growth, 1 for 1–60% take rate, 2 for 60–80% take rate and 3 for 80–100% take rate. Cell lines that overlap two categories are rated at the lower category. For instance, a cell line with 70–90% take rate is rated as category 3. These ratings of tumorigenic potential are denoted “TP.”

### Compound selection

Compounds active against high take-rate cell lines were identified by comparing the growth inhibition measurement (−log GI50) to the four-level rating of take-rate, using Pearson correlation. Thresholds of 0.4 and 0.5 were used to define moderate and strong correlations. Statistical significance was assessed by calculating the expected number of compounds out of all compounds tested that would be expected to have a correlation exceeding a given threshold by chance (based on applying Fisher's Z-transformation and using a standard normal reference distribution).

### Gene expression analysis

Compounds active against cell lines that express relatively high levels of PROML1 or CD44-CD24 were identified using Pearson correlation coefficients between −log_10_GI50 and either log scale expression of PROML1, or the difference between log scale expression levels of CD44 and CD24. PROML1 is represented by a unique probeset on both platforms, CD44 is represented by two U95A probesets and by six U133A/B probesets, and CD24 is represented by one U95A probeset and by six U133A/B probesets. When multiple probesets are available, all are analyzed separately, and differences among all pairs of CD44/CD24 probesets are analyzed separately. For all analyses, compounds for which fewer than 50 cell lines had a GI50 value, or which had no variability in their GI50 values, were excluded from our analysis.

### Standard anticancer agents

A set of 168 compounds with anticancer activity was compiled, and a subset of 121 of them was annotated according to their presumed mechanism of action [8]–[10]. The data we used were obtained from http://dtp.nci.nih.gov/docs/cancer/searches/standard_mechanism_list.html, and include the following mechanism of action classes and numbers of unique structures: alkylating agents (35), antimitotic agents (13), topoisomerase 1 inhibitors (24), topoisomerase II inhibitors (15), DNA anti-metabolites (16), and RNA/DNA anti-metabolites (18).

### Chemical structure comparisons

All compounds discussed here are part of PubChem, and all reported structural comparisons are based on Tanimoto coefficients using the 881 key CACTVS fingerprints. Calculations of Tanimoto coefficients and hierarchical clustering of chemical structures based on Tanimoto coefficients was done using the NCBI portal to PubChem (http://pubchem.ncbi.nlm.nih.gov). We converted all compound identifiers from the DTP's NSC identifier to PubChem's CID identifier for structural analysis.

### COMPARE analysis

COMPARE computations for all of the potent compounds against standard anticancer agents from various mechanistic classes are performed. Pearson correlation coefficients corresponding to high concentration of 0.0001 M are reported for a majority of the compounds. For compounds that are tested with an alternative high concentration, the Pearson correlation coefficients are obtained from pairs with the closest high concentration.

### Selectivity window

The selectivity window was calculated by taking the difference between the average −logGI50 of the most tumorigenic cell lines (take-rate category 3) and the least tumorigenic cell lines (take-rate category 0).

## References

[1] Reya T, Morrison SJ, Clarke MF, Weissman IL (2001). Stem cells, cancer, and cancer stem cells.. Nature.

[2] Al-Hajj M, Wicha MS, Benito-Hernandez A, Morison SJ, Clarke MF (2003). Prospective identification of tumorigenic breast cancer cells.. Proc Natl Acad Sci USA.

[3] Ricci-Vitini L, Lombardi DG, Pilozzi E, Biffoni M, Todardo M (2007). Identification and expression of human colon cancer-initiating cells.. Nature.

[4] Singh SK, Hawkins C, Clarke ID, Squire JA, Bayani J (2004). Identification of human brain tumor initiating cells.. Nature.

[5] Monga M, Sausville EA (2002). Developmental Therapeutics Program at the NCI: molecular target and drug discovery process.. Leukemia.

[6] Teicher BA (1997). *Anticancer Drug Development Guide: Preclinical Screening, Clinical Trials, and Approval;* Eds.

[7] Shoemaker RH (2006). The NCI60 human tumour cell line anticancer drug screen.. Nature reviews.

[8] Boyd MR, DeVita VT, Hellman S, Rosenber SA (1989). Cancer: Principles and Practive of Oncology.

[9] Weinstein JN, Kohn KW, Grever MR, Viswanadhan VN, Rubinstein LV (1992). Neural computing in cancer drug development: Predicting mechanism of action.. Science.

[10] van Osdol WW, Myers TG, Paull KD, Kohn KW, Weinstein JN (1994). Use of the Kohonean self-organizing map to study the mechanism of action of chemotheapeutic agents.. Journal of National Cancer Institute.

[11] Chen K, Kuo SC, Hsieh MC, Mauger A, Lin CM (1997). Antitumor agents. 174, 2′,3′,4′,5,6,7-Substituted 2-Phynyl-1,8-naphthyridin-4-ones: Their Sysnthesis, Cytotoxicity, and Inhibition of Tubulin Polymerimerization.. Journal of Medicinal Chemistry.

[12] Chen K, Kuo SC, Hsieh MC, Mauger A, Lin CM (1997). Antitumor agents. 178. Synthesis and Biological Evaluation of Substituted 2-Aryl-1,8-naphthyridin-4(1*H*)-ones as Antitumor Agents that Inhibit Tublulin Polymerization.. Journal of Medicinal Chemistry.

[13] Kou SC, Lee HZ, Juang JP, Lin YT, Wu TS (1993). Synthesis and Cytotoxicity of 1, 6, 7, 8-Substituted 2-(4′-Subsituted phenyl)-4-quinolones and Related compounds: Identificatin as Antimitotic Agents Interacting with Tubulin.. Journal of Medicinal Chemistry.

[14] Li L, Wang HK, Kuo SC, Wu TS, Lednicer D (1994). Antitumor agents. 150. 2′,3′,4′,5′,5,6,7-Substituted 2-Phenyl-4-quinolones and Related Compounds: Their Synthesis, Cytotoxicity, and Inhibition of Tubulin Polymerization.. Journal of Medical Chemistry.

[15] Li L, Wang HK, Kuo SC, Wu TS, Mauger A, Lin CM (1994). Antitumor Agents 155. Synthesis and Biological Evaluation of 3′,6,7-Substituted 2-Phynyl-4-quinolones as Antimicrotubule Agents.. Journal of Medicinal Chemistry.

[16] Xia Y, Yang ZY, Xia P, Bastow KF, Tachibana Y (1998). Antitumor Agents. 181. Synthesis and Biological Evaluation of 6,7,2′,3′,4′-Substituted-1,2,3,4-tetrahydro-2-phenyl-4-quinolones as a New Class of Antimitotic Antitumor Agents.. Journal of Medicinal Chemistry.

[17] Xia Y, Yang ZY, Xia P, Hackl T, Hamel E, Mauger A (2001). Antitumor Agents. 211. Fluorinated 2-Phenyl-4-quinolone Derivates as Antimitotic Antitumor Agents.. Journal of Medicinal Chemistry.

[18] Pettit GR, Singh SB, Boyd MR, Hamel E, Pettit RK (1995). Antineoplastic Agents. 291. Isolation and Synthesis of Combretastatins A-4, A-5, and A-6.. Journal of Medicinal Chemistry.

[19] Paul KD, Shoemaker RH, Hodes L, Monks AP, Scudiero DA (1989). Display and analysis of patterns of differential acticity of drugs against tumor cell lines: Development of mean graph and COMPARE algorithm.. J Natl Cancer Inst.

[20] Hammond JW, Cai D, Verhey KJ (2008). Tubulin modifications and their cellular functions.. Current Opinion in Cell Biology.

[21] Verhey KJ, Gaertig J (2007). The Tubulin Code.. Cell Cycle.

[22] Westermann S, Weber K (2003). Post-translational modifications regulate microtubule function.. Nat Rev Mol Cell Biol.

[23] Schatten G, Simerly C, Asai DJ, Szoke E, Kooke P, Schatten H (1986). Acetylated a-Tubulin in Microtubules during Mouse Fertilization and Early Development.. Developmental Biology.

[24] Warn RM, Harrison A, Planques V, Robert-Nicould N, Wehland J (1990). Distribution of Microtubules Containing Post-Translationally Modified a-Tubulin During Drosophila Embryogenesis.. Cell Motility and the Cytoskeleton.

[25] Wilson PG, Zheng Y, Oakley CE, Oakley BR, Borisy GG (1997). Differential Expression of Two g-Tubulin Isoforms during Gametogenesis and Development in Drosophila.. Developmental Biology.

